# Range-Expansion in Processionary Moths and Biological Control

**DOI:** 10.3390/insects11050267

**Published:** 2020-04-28

**Authors:** Jetske G. de Boer, Jeffrey A. Harvey

**Affiliations:** 1 Department of Terrestrial Ecology, Netherlands Institute of Ecology, Droevendaalsesteeg 10, 6708 PB Wageningen, The Netherlands; j.harvey@nioo.knaw.nl; 2 Department of Ecological Sciences, Section Animal Ecology, VU University Amsterdam, De Boelelaan 1085, 1081 HV Amsterdam, The Netherlands

**Keywords:** enemy-release hypothesis, invasion, global change, Lepidoptera, forest, public health, urticating setae, pest insect, biodiversity

## Abstract

Global climate change is resulting in a wide range of biotic responses, including changes in diel activity and seasonal phenology patterns, range shifts polewards in each hemisphere and/or to higher elevations, and altered intensity and frequency of interactions between species in ecosystems. Oak (*Thaumetopoea processionea*) and pine (*T. pityocampa*) processionary moths (hereafter OPM and PPM, respectively) are thermophilic species that are native to central and southern Europe. The larvae of both species are gregarious and produce large silken ‘nests’ that they use to congregate when not feeding. During outbreaks, processionary caterpillars are capable of stripping foliage from their food plants (oak and pine trees), generating considerable economic damage. Moreover, the third to last instar caterpillars of both species produce copious hairs as a means of defence against natural enemies, including both vertebrate and invertebrate predators, and parasitoids. These hairs contain the toxin thaumetopoein that causes strong allergic reactions when it comes into contact with human skin or other membranes. In response to a warming climate, PPM is expanding its range northwards, while OPM outbreaks are increasing in frequency and intensity, particularly in northern Germany, the Netherlands, and southern U.K., where it was either absent or rare previously. Here, we discuss how warming and escape from co-evolved natural enemies has benefitted both species, and suggest possible strategies for biological control.

## 1. Introduction

### 1.1. Biodiversity in a Changing World

The field of invasion ecology is an important component of global change biology [1]. In the past, humans have accidentally or deliberately introduced an enormous number of novel plant and animal species to new ecosystems and habitats across much of the biosphere. Although the vast majority remain innocuous, or are even beneficial in their new ranges, acting as alternate food plants, pollinators or predators [2], others have become so abundant that they become ecologically disruptive pests [3,4]. Invasive species may have numerous effects in their new habitats, such as causing the local extinction of native species, disrupting trophic interactions, simplifying food webs, negatively affecting ecosystem functions and disrupting agro-ecosystems [5,6]. The economic costs of harmful invasive species in the United States alone was recently calculated to be at least 120 billion dollars per year [7], and across the world it is considerably more than that. 

Most classical studies of biological invasions have focused on species that are introduced from one continent to another, often surpassing natural barriers such as mountain ranges and oceans, which impede their ability to colonize new and potentially suitable habitats considerable distances from where they naturally occur [3]. A small subset of invasive species become serious pests in their new habitats, disrupting ecosystems and altering the structure of food webs and communities, and in the process driving local declines (or even extinctions) in native species. Several hypotheses have been proposed to explain the remarkable success of invasive species. Three of the most pervasive of these are the ‘enemy-release hypothesis’ (ERH) [8], the ‘novel weapons hypothesis’ (NWH) [9], and the ‘evolution of increased competitive ability hypothesis’ (EICA) [10]. The three hypotheses are not mutually exclusive but are closely correlated. For instance, the ERH predicts that invasive species escape from their co-evolved natural enemies from their original range, often because they possess novel traits such as defence (NWH), and as a result can re-allocate limited metabolic energy away from defence towards growth or reproduction (EICA). Numerous studies have shown that plants and animals that successfully establish in new habitats possess novel traits, such as secondary metabolites in plants, or high reproductive potential in animals, that enable them to out-compete native species [2,11,12,13]. 

### 1.2. Climate Change and Range Expansions: A Different Type of Invasiveness

In the last 40 years, the biosphere has been warming rapidly, largely due to anthropogenic factors, such as the indiscriminate burning of fossil fuels, which is generating increases in atmospheric concentrations of greenhouse gases, especially carbon dioxide [14,15]. Anthropogenic global warming (AGW) is also exacerbated by forest clearing and other changes in land use [16]. Moreover, most scientists agree that the frequency of extremely warm years and short-term extreme climate events (ECEs) such as heatwaves, droughts, cloudbursts and attendant processes like floods and fire will also continue to increase in the coming years [17,18]. 

The effects of AGW on species, multitrophic interactions and ecosystems have received considerable attention in the last twenty years [19,20,21]. The general conclusion is that it has had a negative effect on biodiversity at all levels of organization because warming and ECEs are pushing some species to (or even beyond) their adaptive limits. However, other studies also suggest that warming, at least initially, will benefit thermophilic insects, increasing the risk of pest outbreaks [22,23,24,25,26]. Insect diversity and abundance generally increases towards lower latitudes, and as the planet warms, many more species found in southerly regions will move polewards in the north temperate regions, including pests of agriculture, insects that transmit diseases and pests that infest towns, parks and even urban centers. For example, warming has clearly benefitted the diamondback moth (*Plutella xylostella*), which is considered to be the most serious pest of collard and mustard crops in the world [27]. This species originates in North Africa and the Mediterranean region but has since been introduced widely. In central Europe and North America, cold winters used to kill overwintering stages, but recent warming—in particular, higher minimal temperatures at night, attendant with a reduction in severe winter frosts—has enabled *P. xylostella* to survive as far north as southern Britain and Canada, and it continues to overwinter successfully polewards with continued warming [27,28,29]. Winter and cold weather are considered as important abiotic factors in pest control, and warmer winters will likely allow many pest species currently found in warmer temperate regions to survive farther and farther northwards over time. 

Although increased overwintering survival will undoubtedly affect the temporal and spatial dynamics of pest outbreaks, range expansions may also affect other important processes such as seasonal phenology and interactions with both mutualists and antagonists. If the specialist (co-evolved) natural enemies of a range expanding herbivore ‘remain behind’, and therefore do not track it to its new range, this can act as a means of enemy-release and allow the herbivore to become a serious pest in new habitats where it establishes. In the case of *P. xylostella*, its largely specialized parasitoids have apparently tracked its poleward and/or elevational range shifts [27] and some of its more thermophilic parasitoids are becoming more abundant in northern Europe [30]. However, other insects have become pests in part because of climate-change mediated range expansions and concomitant escape from some of their natural enemies [24,31,32,33,34]. In the following sections, we review the effects of climate change on processionary moths and their natural enemies, and discuss the enemy-release hypothesis with respect to outbreaks of these species in areas outside their core range. We then evaluate potential strategies for the biological control of processionary moths and conclude with a research agenda. While our focus is on oak processionary moth, we also discuss pine processionary moth because it is much better studied than any other *Thaumetopoea* species, and much can be learned from studies on its population dynamics and the role of natural enemies. 

## 2. Processionary Moths

**Figure 1:**
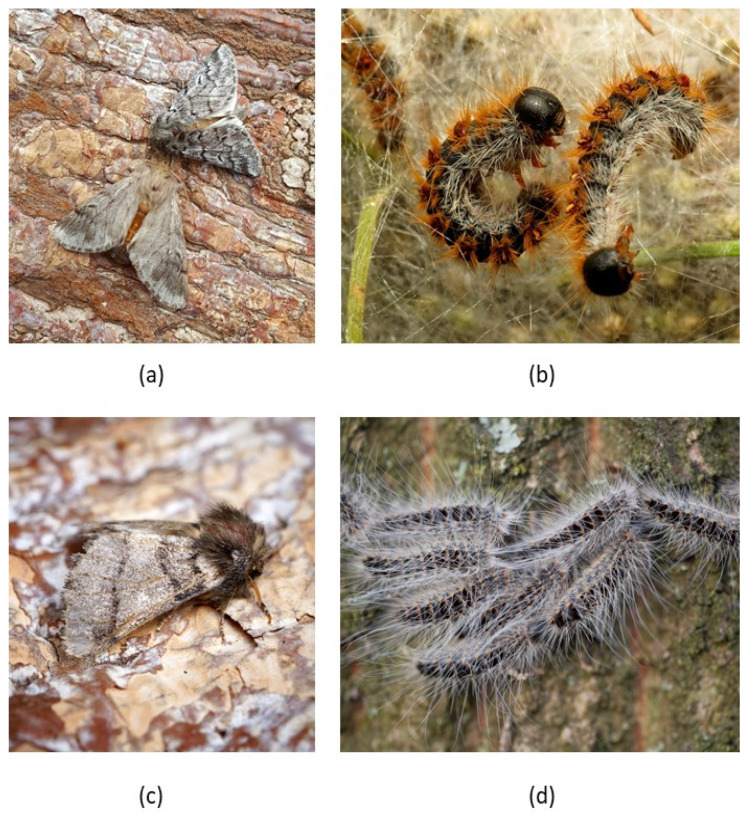
Pine processionary moths (PPM) and oak processionary moths (OPM) in their natural habitat. (**a**) A pair of PPM adults on the bark of a tree; (**b**) PPM caterpillars on their silk nest amongst pine needles; (**c**) OPM adult on a tree; (**d**) Procession of OPM caterpillars on the bark of an oak tree. All images were retrieved from Wikimedia Commons; images are licensed under the terms of the CC-BY-2.0 (**a**–**c**) or CC-BY-SA-4.0 (**d**). (photos a and c, Ben Sale; b, Katja Schulz; d, Luc Hoogenstein).

Processionary moths (Notodontidae: Thaumetopoeinae) are well known for the behavior of their larvae. Caterpillars are gregarious throughout their development: they aggregate in ‘nests’ and typically move in lines or groups to forage or when they are ready to pupate. The genus *Thaumetopoea* consists of about 15 species that are native to Europe, the Middle East and Northern Africa [35,36]. Of these, the pine processionary moth (PPM, *T. pityocampa*) is the best-studied species because it is a severe defoliating pest of pine and cedar trees in Europe [37,38]. Oak processionary moth (OPM, *T. processionea*) is also well known, particularly because it has become a pest in the Benelux region and neighboring countries in the last 30 years [39,40], where it increasingly causes public health problems (Figure 1).

### 2.1. Life History and Pest Status of Oak and Pine Processionary Moths

Both the PPM and OPM are univoltine and strongly associated with their host trees. PPM is oligophagous on native and introduced species of *Pinus* and *Cedrus* [37]. PPM females lay eggs in clusters on pine needles or twigs early in summer and eggs hatch at the end of summer, although this depends on latitude and altitude [37]. Caterpillars feed during winter, and their foraging activity and development rate thus largely depend on winter (night) temperatures [37]. The only other *Thaumetopoea* species that feeds during winter is the eastern pine processionary moth *T. wilkinsoni*. Mature caterpillars of PPM pupate in the soil in spring, with new adults typically emerging a few months later, although prolonged diapause also occurs, with adults emerging one or more years later [41]. 

OPM females lay egg clusters on small branches in the upper canopy of oak trees (*Quercus* species) in summer. First instar larvae within eggs develop fully by the end of autumn. These larvae are highly cold-tolerant—they can survive temperatures of −30 °C—and overwinter as embryos inside the egg to hatch the following spring. Young OPM caterpillars can go without food for several weeks, making them well-adapted to early hatching relative to oak budburst [42]. Mature OPM caterpillars pupate in nests on the trunk of their host tree where adults emerge in late summer.

Processionary caterpillars weave silken nests containing excuviae, hairs (including setae) and excrement. PPM nests are usually built around one or more smaller branches, while OPM nests can be found on the trunks or thicker branches of a tree, or sometimes at the foot of the trunk. These nests provide shelter for the larvae while they rest and moult, protecting them from (certain) natural enemies. Caterpillars disperse in ‘processions’ to feed during the night and return to the nests before dawn to avoid diurnal foraging natural enemies. Individual nests can contain many hundreds or even thousands of larvae. As mentioned, PPM is one of the major pests of pine stands in Europe and it has therefore received considerably more attention than other *Thaumetopoea* species. High densities of OPM can also cause substantial damage to the common oak (*Quercus robur*) [43] and, to a lesser extent, other related oak species. If an individual tree is defoliated, the larvae will abandon their nest during the night and crawl in a lengthy procession to a new host tree, where they construct a new nest. Individual trees can harbour between 10,000 and 100,000 caterpillars [39]. 

The recent attention for processionary moths relates to their impact on human health. From the third instar, caterpillars of PPM and OPM produce urticating hairs that contain the toxin thaumetopoein [36,37,44]. When caterpillars are disturbed or threatened, they release these setae into the environment [45]. This is one of the caterpillars’ defence mechanisms against their natural enemies. However, when PPM or OPM hairs come into contact with human skin or other membranes, they lead to serious irritation and, in extreme cases, can even cause blindness, thus posing a serious threat to human and animal health [36,45]. This effect contributes greatly to the current pest status of OPM because oak trees are often present in or close to urban environments and humans are more likely to encounter (hairs of) OPM than PPM [39,43].

### 2.2. Distribution, Population Dynamics and Effects of Climate Change on Range Expansion

PPM is native to the Mediterranean basin, with its distribution currently ranging from North Africa to central Europe. The OPM has a slightly more northwards native distribution than the PPM and its current distribution ranges from the middle-east to north-western Europe [39,46]. Host trees are present beyond the edges of the current ranges of both species and do not appear to limit range expansion, although local differences in tree defence chemistry may influence their suitability for processionary moths, and thus influence the speed of range expansion. Climatic factors, such as warming, may differentially influence processionary moths [37], with winter-active species being affected more because winter temperatures are relatively more increased [47]. Indeed, there is clear evidence that PPM is expanding its range and is able to establish at higher altitudes in response to climate warming [36,37,47,48]. The species has apparently traversed the Alps and is expanding northwards into northern France, where transplanted pines from the South have allowed for the establishment of pioneer populations beyond the range limit [32]. The picture is less clear for OPM. Historical data show that the species was present, although not abundant, in much of its present range already before 1920, and provide no evidence for long-term longitudinal range expansion in response to warming [39]. Surprisingly, OPM was rare or even absent in the many northern areas for close to a century [39]. OPM was rediscovered in Belgium in 1971, and it reinvaded the Netherlands in south Limburg in 1991 [43], thereafter rapidly spreading northwards, reaching northern Dutch provinces by around 2000 [44]. As of this writing, it is perhaps more abundant locally in parts of the Netherlands than anywhere else in its present range [37,39]. Several factors, including climate warming and land-use change, have contributed to the current pest status of the OPM [39]. As for PPM, human-mediated transport may also accelerate the range expansion of OPM, for example in its establishment in the United Kingdom in 2006 [49,50].

Like many forest pests, PPM and OPM have outbreaks, during which very high densities can be reached, interspersed with periods of low abundance. The drivers of these population cycles are not well understood due to a scarcity of long-term studies, which are sometimes complicated or disrupted by pest control and forest management practices [37]. Various negative density-dependent mechanisms likely play a role in regulating populations of processionary moths, for example a reduction in host quality in response to high caterpillar densities, possibly in combination with entomopathogens. Climatic factors also contribute to local population dynamics although perhaps not in the same way as they influence range expansion. Indeed, the population growth rate of PPM in the Southern Alps was affected most by summer temperatures and rainfall and not by winter temperatures [51], while dry conditions in spring were associated with high densities of OPM in Hungary [52]. Favourable annual weather conditions also likely contributed to the high densities of OPM in recolonized areas in the Benelux region and neighbouring countries such as Germany and the United Kingdom in the last 30 years [39,40]. In turn, high densities at the edge of the range increase the number of dispersing adults, accelerating range expansion. Identifying the factors that drive population dynamics and range expansion in processionary moths is an important research topic because the current lack of knowledge hinders the prediction of the response of these species to climate change and how they develop as pests in the future. Importantly, models for range expansion of herbivores should not only consider the climate and the presence of host plants, but should also take into account interactions with natural enemies. 

### 2.3. Natural Enemies of the OPM and PPM

Processionary moths are well defended against natural enemies during all life stages. Eggs are laid in clusters that are covered by the hairs and scales of the mother. Caterpillars aggregate in nests covered with a dense layer of silk and hairs during the day to minimize attack by diurnal natural enemies (Figure 1b). Third to last instar caterpillars produce urticating hairs that likely evolved as defence against (generalist) vertebrate predators) [45]. Pupation occurs in in the soil for PPM, while OPM pupates in the nest on the trunk of the host tree [37]. Adults are well camouflaged (Figure 1a). Nevertheless, all life stages of *Thaumetopoea* have natural enemies in nature, again with most scientific records and studies being available for PPM. Unfortunately, little specific information is available for other processionary moths, including OPM, although natural enemies are partially shared across species [37]. This is particularly true for generalist predators, such as birds, ants and beetles, and generalist pathogens such as entomopathogenic fungi. This is less evident for parasitoids because they are often more specialized to their host. Nevertheless, some parasitoid species can reproduce on multiple species of processionary moths. For example, the tachinid parasitoid *Blondelia pinivorae* occurs widely across Europe, parasitizing northern PPM *T. pinivora* in Sweden and Spain and the Eastern cedar processionary moth *T. ispartensis* in Turkey [53]. Here, we summarize the scientific knowledge on natural enemies of *Thaumetopoea* species, highlighting potential differences between PPM and OPM.

Insectivorous birds and bats are efficient predators that are known to respond to spatial and temporal fluctuations of their prey. Abundance of the great tit (*Parus major*) increases with PPM density in south-eastern France [54]. Some bird species are adapted to feeding on caterpillars with urticating setae, for example through a special gizzard wall structure (cuckoo species) or by eating only the inner parts of caterpillars (tit species). Other birds are specialized predators of soil-borne pupae of PPM, such as the Eurasian hoopoe *Upupa epops* [55]. Various bird and bat species have also been observed to prey on all life stages of OPM, although scientific studies are lacking. We expect that the role of these vertebrate predators in population regulation of OPM may be different from that of PPM because of their different life cycles. PPM caterpillars are available in winter when few other prey are present, while OPM caterpillars are present in summer when many other caterpillar species are available to foraging birds.

**Figure 2:**
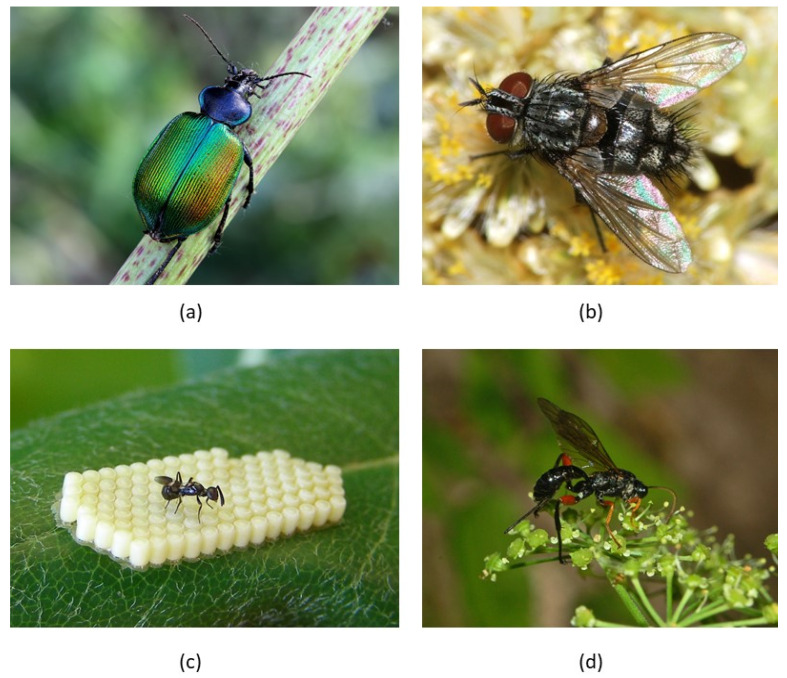
Natural enemies of processionary moths that may play a role in preventing or reducing population outbreaks. (**a**) The forest caterpillar hunter *Calosoma sycophanta*. This carabid beetle was introduced in North America to control gypsy moth, and is also a voracious predator of PPM and OPM caterpillars; (**b**) A representative species of the genus *Carcelia*. The tachinid parasitoid fly *C. iliaca* could be a promising biocontrol agent of OPM; (**c**) The egg parasitoid *Anastatus bifasciatus* on an egg mass of *Nezara* bugs. Many species of hymenopteran parasitoids are found on PPM and OPM eggs and some species may be suitable for classical biological control introductions; (**d**) *Pimpla* sp. female foraging for nectar. Considering the environmental needs of natural enemies is an essential component of conservation biological control. Images are licensed under the terms of the CC-BY-2.0 (**a**) or CC-BY-SA-3.0 (**b**–**d**). (photo a, Anatoly Mikhaltsov; b, Rui Andrade; c, H. Dumas; d, Hectonichus).

Many invertebrate predators have been reported in various stages and species of processionary moths. For example, 21 generalist predators were observed to prey on Eastern PPM *T. wilkinsoni* in Israel, most of which were insects or spiders [56]. Caterpillars are prey for several beetle and bug species [37], of which *Calosoma sycophanta* and *C. inquisitor* may be the most important (Figure 2). The adults and larvae of these carabid beetles feed on caterpillars and pupae and are often found in trees. *Calosoma sycophanta*, known as the forest caterpillar hunter, has been studied as a natural enemy of the gypsy moth (*Lymantria dispar*) particularly [57] but also as a predator of PPM in Turkey [58] (see below). 

*Villa brunnea* is the only dipteran parasitoid of the Bombyliidae family found in southern Europe and parasitizes PPM pupae at highly variable rates [59]. In contrast, dipteran parasitoids of the families Tachinidae form a highly diverse, and possibly important, group of natural enemies of processionary moths [37]. These flies are larval or larval-pupal parasitoids with diverse life histories, including specialists (on one or a few *Thaumetopoea* species) and species with a broad host range, species that oviposit macrotype eggs on the host and species that lay microtype eggs in the host’s habitat. Several studies showed that *Phryxe caudata* is the most prevalent parasitoid of PPM and it can be found consistently across years and sites, but parasitism rates are often less than 5% [59,60,61]. The host range of this tachinid fly is probably narrow and it produces two generations per year, the first one in PPM caterpillars and the second one in the pupae [62]. *Carcelia iliaca* is a specialist tachinid parasitoid of OPM caterpillars (Figure 2), known from many countries, including Turkey, Romania, France, the Netherlands and the United Kingdom [37,63,64,65]. Parasitism rates have not been recorded recently in most of the geographic range of OPM, with the exception of a molecular study in the United Kingdom. Almost half of all collected late instar OPM caterpillars were found to be parasitized by *C. iliaca*, whereas a few caterpillars were parasitized by the generalist tachinid *Compsilura concinnata* [50].

A wide diversity of hymenopteran parasitoids uses processionary moths as hosts. Of these, egg parasitoids have been recorded most often and are best studied, particularly those of PPM. *Baryscapus servadeii* (Eulophidae) and *Ooencyrtus pityocampae* (Encyrtidae) are the most reported specialist and generalist egg parasitoids of PPM eggs, respectively [37], the latter requiring an alternative host species to complete its first generation before PPM eggs are available. *Anastatus* cf. *bifasciatus* (Eupelmidae) is a generalist egg parasitoid that is found regularly on OPM eggs, for example in Italy and Germany [66,67]. Different *Thaumetopoea* species appear to be differentially susceptible to egg parasitism. For example, egg parasitism rates were 2–7 times higher in PPM than in eggs of the Pistachio processionary moth *T. solitaria* in the same region in Bulgaria [68]. Many other factors contribute to egg parasitism rates, including temperature, altitude, and even the presence of hyperparasitoids [32,69,70,71]. The proportion of parasitized egg clusters, as well as the percentages of parasitized eggs within clusters, are indeed highly variable across years and location [32,67,72]. Relatively few hymenopteran parasitoids have been reported in the caterpillars and pupae of processionary moths and they have been studied in much less detail than egg parasitoids or tachinid parasitoids [37]. Examples of larval parasitoids include *Meteorus versicolor* (Braconidae), that is possibly specialized to some extent, and the highly polyphagous *Dibrachys cavus* (Pteromalidae), that is also known as a hyperparasitoid [37]. *Colichneumon rudis* is a specialist ichneumonid parasitoid on PPM pupae [59,60,61], while *Pimpla processioneae* (Ichneumonidae) is a specialist on OPM pupae, which are also parasitized by two generalist *Pimpla* species [73]. To date, no studies have evaluated the impact of larval and pupal parasitoids on the population regulation of processionary moths. It also remains unclear how parasitoids respond to outbreaks of processionary moths, with the exception of a study in southern Italy that showed an inversely density-dependent relationship between pupal parasitism rate and pupal density of PPM [59]. 

In addition to the (in)vertebrate natural enemies reviewed above, entomopathogenic nematodes and other entomopathogens can infect processionary moths [37]. As suggested, this group of mostly generalist natural enemies may (collectively) contribute to the population regulation of processionary moths [51], but little information is available on natural infection rates in most of the following examples. *Steinernema* and *Heterorhabditis* nematodes collected from soil in Turkey or Algeria may infect (Eastern) PPM (pre)pupae [74,75]. Because PPM pupates in the soil, it may more frequently be exposed to entomopathogenic nematodes than OPM. To our knowledge, nematodes on OPM in nature have not been reported in the scientific literature, although formulations are available for biological control (see below). OPM are infected by several microsporidia species in Austria but the virulence and natural infection rates were low [76]. Several species of entomopathogenic fungi (e.g., *Beauveria bassiana*), bacteria (*Bacillus thuringiensi*) and viruses (e.g., *Smithiavirus pityocampae*) can also infect processionary moths [37,59,61,74]. 

### 2.4. Range Expansion of OPM and PPM and the ERH

Clearly, the OPM and PPM harbor many natural enemies in the core of their range, but the effectiveness of these enemies in controlling outbreaks of these pests appears to be limited. Moreover, the success of processionary moths in recently colonized habitats may be due to escape from co-evolved enemies that have not tracked their northwards expansion (the ERH). However, little is known about the impact of natural enemies along latitudinal or longitudinal gradients. Nevertheless, the OPM is not considered a serious pest over much of its native central and southern European range. Although outbreaks occasionally occur, they are generally followed by lower population densities, possibly because its numbers are reduced by specialized natural enemies that have not tracked its northern expansion. This may be because there is no evolutionary incentive for co-evolved enemies of the OPM to track it northwards, at least if there is no density-dependent competition among them for hosts or prey. Under these conditions, natural enemies only slowly disperse over time. For example, the forest caterpillar hunter *C. sycophanta* disperses more slowly than its prey [77]. This carabid beetle used to be present in the Netherlands and Belgium, but is now (nearly) extinct, while the congeneric *C. inquisitor* has also declined significantly in Belgium in recent decades [78]. It appears that *C. sychophanta* is highly susceptible to pesticides [79], which may account for its recent decline and failure to re-establish in parts of its former range, where the OPM is now abundant. Pesticides may also hamper the ability of *C. inquisitor* to respond to OPM outbreaks. 

Several studies on PPM provide support for the ERH, at least for egg parasitoids. For instance, no egg parasitoids were found in pioneer populations of PPM in the Paris basin, in contrast to PPM populations in the core area in France [32]. The tachinid parasitoid *P. caudata* was observed in the new area, possibly because PPM was introduced in the pupal stage that hosts this parasitoid. Prolonged development at lower temperatures in newly colonized areas may further promote parasitism rates of *P. caudata* on PPM [72]. In addition, the Northern PPM *T. pinivora*, which occurs from south-western to northern Europe, shares egg parasitoids with PPM in Spain, but no egg parasitoids have been found in Sweden [37]. Further, PPM egg mortality due to parasitism was lower at a higher altitude [72]. A study in Bulgaria suggests that 21 years after PPM invaded a new area, egg parasitism rates were equal to those in the core area of PPM [71]. *Trichogramma embryophagum* appears to have been the first parasitoid on PPM eggs in this area, while the main egg parasitoids remained absent for a longer time.

To date, no egg parasitoids have been reported on OPM in areas where the species invaded or re-established and is considered a threat. However, a few other specialized natural enemies of OPM appear to track the movements of their host relatively quickly. Two tachinid parasitoids were found in the Netherlands only a few years after OPM re-established: *Pales processionea* in 1991 and *Carcelia iliaca* in 1993. It is therefore expected that their capacity to expand their range along with their host is rather good [64]. Sands et al. [63] described *C. iliaca* as new for the United Kingdom in 2014, only a few years after OPM was established in West London. *Pimpla processioneae* constitutes another example. This specialist pupal parasitoid of OPM was discovered in the Netherlands only a few years after the re-establishment of OPM [73]. The absence of records of *P. processioneae* in the Netherlands in the period that OPM was absent supports the specific interaction between the host and parasitoid, and demonstrates that *P. processionea* tracks populations of its host. 

We conclude that the current pest status of OPM in the northern parts of its range may be explained, in part, by the ERH [8]. However, several specialized natural enemies have followed their host northwards, while generalist vertebrate predators are also present. Other factors likely play a role as well and need to be addressed, including climate, land-use (intensive agriculture, habitat fragmentation) and biodiversity decline. These factors may also explain why novel natural enemies in the new range do not appear to be using OPM, although this has been little studied to date. Such studies are urgently needed to develop efficient control measures in areas where OPM is considered problematic.

## 3. Control of OPM and PPM

The increasing abundance of processionary moths has resulted in various measures by local and national governments to reduce infestation levels, including chemical pesticide and biocide applications, the burning of larval nests and physical removal. However, to date, despite being labour-intensive and costly, the success of these measures has been limited, and some measures likely have substantial effects on non-target (or even endangered) species. We propose two routes to developing sustainable biological control of OPM and PPM in newly colonized areas or regions where it had re-established: (1) classical biological control, where co-evolved specialist natural enemies are mass-reared and released with the intention that they establish in the field; (2) conservation biological control, where the environment is manipulated in such a way that existing populations of natural enemies are enhanced. These two routes are not mutually exclusive because local conditions should be optimized to maximize the benefit of biological control introductions. 

### 3.1. Classical Biological Control

Biological control introductions have been made in the past against (Eastern) PPM, including the egg parasitoid *O. pityocampae* and the tachinid fly *Exorista segregata*. *O. pityocampae* can be reared in the laboratory over many generations, in a range of alternative host eggs, including *Bombyx mori* [56,80], and even artificial eggs [81]. While a substantial increase in egg parasitism rate from 6% to 20% was observed in one year, the number of *O. pityocampae* generations after release may be limited and the success of releases are likely highly dependent on weather conditions such as temperature and rainfall. Nevertheless, introductions may be useful in areas where egg parasitoids are missing or scarce [37], especially because the dispersal capability of these small organisms is thought to be low [32]. Such introductions may be particularly helpful at the front edge of its range or in pioneer populations, where eradication attempts are made. Under these conditions, egg parasitoids could decrease the survival capability of small PPM colonies and thereby slow the spread of range expansion. Egg parasitoids that are commercially available, such as *Trichogramma brassicae* or the congeneric *T. embryophagum*, may also be used for this purpose as they are known to parasitize processionary moth eggs [71,82]. We are not aware of any experimental studies on the potential of specialist OPM egg parasitoids for classical biological control.

The carabid beetles *C. sycophanta* and *C. inquitisor* may be interesting candidates for classical biological control of processionary moths for several reasons. Both species are native in the distribution range of OPM. Adults are long-lived, highly fecund and voracious predators of (hairy) caterpillars and pupae, including OPM and PPM, so they are partially specialized. *Calosoma sycophanta* (Figure 2a) has been studied intensively for biological control of the gypsy moth *L. dispar* (Erebidae) in North America, where it was introduced in the early 20th century [57,83,84]. The species can be reared under laboratory conditions [58,85], and studies in the United States and Canada after its release against *L. dispar* show that it can establish and extend its range when suitable habitat and prey are available. Given their susceptibility to pesticides, however, it is necessary to stress that the effectiveness of both beetles to control OPM and PPM, and recovery from their recent declines may hinge on changes in agricultural practices that often rely on intensive use of chemical control of crop pests [86]. As for egg parasitoids, additional releases of *C. sycophanta* in the early phase of an OPM outbreak may also be useful because the response of the predator to prey abundance is relatively slow [57]. Other candidate natural enemies of OPM include specialist parasitoids, which only form a threat to OPM and not to other native insect species, such as the tachinid *C. iliaca*, but no studies are available to date. 

Microbial biological control of processionary moths is also a possibility [59,87] although we do not discuss this in detail here because only few entomopathogens have a specialist relationship with their insect hosts. Less specific entomopathogens, such as the fungus *B. bassiana* or formulations based on the bacterium *B. thuringiensis*, may be useful in quickly reducing numbers of processionary caterpillars in case of (human) health risks. More specific entomopathogens may exist, for example viruses, such as *Smithiavirus,* that was found to be highly effective against PPM in the middle of the 20th century but not further investigated due to difficulties cultivating the virus [37]. We think this deserves further study because modern techniques may allow for the safe production of such viruses and make this a cost-effective option. Another interesting opportunity may exist in using arthropod natural enemies as vectors of microbial control agents. For example, *C. sycophanta* can transmit microsporidia to gypsy moth caterpillars [88]. It may be possible to enhance the efficiency and specificity of entomopathogenic microbes in this way.

### 3.2. Conservation Biological Control

The effect of existing populations of natural enemies on processionary moths may be enhanced by considering their environmental needs. More diverse plant communities offer a greater availability of alternative prey or hosts as well as other habitat resources to potential natural enemies of processionary moths. The free-flying adults of many parasitoid species feed on floral or extrafloral nectar (Figure 2d), but this resource may not be always available in forests. Alternatively, the longevity of parasitoids can be increased by feeding on carbohydrate-rich aphid honeydew [89]. A higher vegetation diversity leads to a greater availability of honeydew throughout the year. Feeding on honeydew can indeed increase the longevity of two egg parasitoids of PPM (*O. pityocampae* and *B. servadeii*) and this is important because it increases the chance that *O. pityocampae* survives until the oviposition period of PPM [90]. In the same way, a higher vegetation diversity leads to more structural diversity in forests, thus providing more nesting sites for insectivorous birds [91]. Installing man-made nesting sites is also possible and this has been evaluated in France for the management of PPM. Nest boxes were found to be gradually utilized by great tits but the effect on numbers of PPM tents was highly variable across years (and sites) and it remained difficult to draw conclusions after 5–7 years of monitoring [37]. Because results may depend on local conditions, further studies on this approach are needed, for example on its effect on regulating OPM numbers in the Benelux region.

Besides generating positive effects on natural enemies, there are likely other benefits of increasing vegetation diversity as well. Tree diversity can contribute to pest reduction in forests [92], and several mechanisms may reduce infestation by PPM in mixed forests. For example, host finding may be influenced by the masking of infochemicals used to locate host trees [93] or the production of repellent compounds such as methyl salicylate [94]. However, the contribution of each of these mechanisms is difficult to assess and few studies have quantified the effects. Furthermore, whether such mechanisms play a role in OPM population densities has not yet been investigated. 

We see the conservation biological control of PPM and OPM as an important area of research because this approach is needed to transition from short-term control of pest outbreaks to a management strategy with long-term effects and that prevents outbreaks. In order to achieve this, we need to better understand the environmental requirements of natural enemies, in particular parasitoids, of processionary moths and their ecological niches. At the same time, conservation biological control will benefit from an increased understanding of how land-use (e.g., intensive agriculture) and climate influence the presence of parasitoids and predatory natural enemies of processionary moths.

## 4. Conclusions and Research Agenda

Processionary moths are causing increasing health problems, driven by climate warming, changes in land-use and the increased contact-risks with the urticating hairs of processionary caterpillars in densely populated countries such as the Netherlands. In their core range, *Thaumetopoea* populations may also reach high densities but these outbreaks are typically cyclic and intermittent with periods of lower population densities. It is not completely clear how the broad suite of natural enemies responds to outbreaks and what their role is in population regulation of *Thaumetopoea* species. However, it is notable that population densities of OPM have not gone down and outbreaks have continued to occur and increase in frequency in re-invaded areas. Studying population dynamics in newly invaded areas, in particular in relation to climatic factors [52], is needed to predict how processionary moths will expand their range further and develop as pests in the future. To date, few studies have investigated the presence of natural enemies of OPM and PPM, specifically in recently (re-)invaded areas [32,50,64,71]. Studies comparing natural enemies along a longitudinal gradient are urgently needed but are often complicated by difficulties handling species that can cause human health problems. Molecular methods may therefore be an important tool in such studies, for example the nested metabarcoding on OPM caterpillars to investigate the suite of natural enemies in a population in West London [50]. In this study, arthropod CoxI primers were used, but it would be possible to extend this method to fungal natural enemies by using fungal ITS primers. 

Other questions that need to be addressed are: how do climate warming and land-use change influence OPM and PPM parasitoids and their host-tracking? What are the host preferences and host location mechanisms of potential generalist natural enemies that are already present at the front edge of the range of processionary moths? Can populations of such existing natural enemies be enhanced through manipulating the environment? Finally, classical biological control introductions may be used to manage population outbreaks of processionary moths, specifically to slow the spread of range expansion of PPM and to reduce the frequency and intensity of OPM outbreaks. Such strategies require precise local knowledge of the target pest insect and its phenology. Non-target risks of biological control introductions should be carefully evaluated because natural enemies are often able to use alternative host or prey species. Addressing these questions on the natural top-down control of processionary moths is necessary to predict the future range expansion and pest status of OPM and PPM. Moreover, this knowledge is essential to change from current short-term control of processionary moth outbreaks, with potentially severe non-target effects, to a management strategy that is sustainable in the long-term and may even positively influence biodiversity in the environment where OPM and PPM currently thrive.
